# Medial elbow approach via a medial epicondyle osteotomy

**DOI:** 10.1007/s00064-026-00953-w

**Published:** 2026-09-11

**Authors:** John M. Ibrahim, Bryan J. M. van de Wall, Christian Michelitsch, Korbinian Perl, Reto Babst, Frank J. P. Beeres

**Affiliations:** 1 Department of Orthopedics, University of Pittsburgh Medical Center, Pittsburgh, PA USA; 2 Klinik für Orthopädie und Unfallchirurgie, Luzerner Kantonsspital, Spitalstrasse, 6000 Luzern, Switzerland; 3 Faculty of Health Science and Medicine, University of Luzern, Luzern, Switzerland; 4 Unfallchirurgie, Kantonsspital Graubünden, Chur, Switzerland

**Keywords:** Coronoid fracture, Terrible triad, Complex elbow, Posteromedial instability, Extended medial, Koronoidfraktur, Terrible Triad, Osteotomie des medialen Epikondylus, Medialer Ellenbogenzugang, Posteromediale Rotationsinstabilität

## Abstract

**Objective:**

To describe a medial epicondyle osteotomy technique to approach the coronoid from the medial side and to present the outcome of our patients.

**Indications:**

Displaced coronoid fractures (particularly O’Driscoll anteromedial subtypes 1–3 and basal subtypes 1 and 2 fractures).

**Contraindications:**

Infected or compromised skin on the medial elbow.

**Surgical technique:**

A curvilinear incision centered over the anterior aspect of the medial epicondyle is performed. The ulnar nerve is released. The medial epicondyle is osteotomized in a trajectory that parallels both the medial trochlea and the fibers of the flexor–pronator mass, from posterior to anterior. This is performed with a sagittal saw and completed with an osteotome. The flexor pronator mass and medial ulnar collateral ligament (MUCL) are reflected distally and the anterior capsule is elevated to expose the articular surface of the ulnohumeral joint. The coronoid fracture is anatomically reduced and fixed with either (headless) compression screws or a (anatomic) buttress plate, depending on the fracture configuration. The medial epicondyle is reduced and fixed in place with two diverging cortical screws (2.7 or 3.5 mm). A stable construct should be achieved to allow for early mobilization.

**Postoperative management:**

Free range of motion exercises can be initiated immediately. At 6 weeks, strength exercises can be initiated. At 12 weeks, the patient may return to activities as tolerated.

**Results:**

In all, 13 patients (7 men and 6 women; ages 20–62 years old) underwent fixation of their complex coronoid fractures through a medial epicondyle osteotomy. Average elbow flexion–extension arc of motion was 118° and average pronation–supination arc of motion was 154°. One complication was reported in a patient who required revision fixation of the medial epicondyle osteotomy.

## Abstract (de)

**Operationsziel:**

Beschreibung einer Osteomietechnik des medialen Epikondylus zur Darstellung der medialen Seite des Koronoids und Outcome unserer Patienten.

**Indikationen:**

Dislozierte Koronoidfrakturen (insbesondere anteromediale Subtypen 1–3 nach O’Driscoll und basale Frakturen vom Subtyp 1 und 2 ).

**Kontraindikationen:**

Infizierte oder geschädigte Haut am medialen Ellenbogen.

**Operationstechnik:**

Durchführen einer geschwungenen Inzision zentral über dem anterioren Aspekt des medialen Epikondylus. Der N. ulnaris wird freigelegt. Der mediale Epikondylus wird in einer Parallelen zur medialen Trochlea und den Fasern der Flexoren sowie des Pronator teres von posterior nach anterior osteotomiert. Dies wird mit einer oszillierenden Säge durchgeführt und mit einem Osteotom abgeschlossen. Die Flexoren-Pronator-Masse und das mediale ulnare Kollateralband (MUCL) werden nach distal weggeklappt und die vordere Kapsel wird angehoben, um die Gelenkfläche des ulnohumeralen Gelenks freizulegen. Die Koronoidfraktur wird anatomisch reponiert und je nach Frakturmuster mit (kopflosen) Kompressionsschrauben oder einer (anatomischen) Abstützplatte fixiert. Der mediale Epikondylus wird anatomisch reponiert und mit zwei divergierenden Kortikalisschrauben (2,7 oder 3,5 mm) in Zugschraubentechnik fixiert. Es sollte ein stabiles Konstrukt erreicht werden, um eine frühe Mobilisation zu ermöglichen.

**Weiterbehandlung:**

Eine funktionelle, belastungsfreie Mobilisation kann unmittelbar postoperativ begonnen werden. Nach 6 Wochen erfolgt der sukzessive Kraftaufbau. Zwölf Wochen postoperativ ist eine Vollbelastung, soweit tolerierbar, erlaubt.

**Ergebnisse:**

Insgesamt 13 Patient:innen (7 männlich, 6 weiblich; Alter 20–62 Jahre) erhielten eine Rekonstruktion ihrer komplexen Koronoidfraktur durch eine mediale Osteotomie des Epikondylus. Der durchschnittliche Bewegungsumfang (Flexion – Extension/Pronation – Supination) betrug 118° respektive 154°. Es trat eine Komplikation bei einem Patienten auf, welcher eine Revision der Fixierung der medialen Epikondylusosteotomie benötigte.

## Introductory remarks

Several anatomic approaches exist to expose the medial aspect of the elbow. Huang et al. provide an anatomic comparison of the exposure granted by five different soft tissue approaches to the medial elbow [1]. These approaches are often used to treat fractures of the proximal ulna which most commonly involve the coronoid, fractures of the distal humerus, as well as for ligamentous pathology of the medial elbow [2–4]. For coronoid fractures in particular, the approach should ideally provide visualization of the joint as these fractures are often intra-articular, and it should provide adequate exposure of the bony surface of the coronoid for anatomic reduction and implant placement for fixation of the fracture [5]. Additionally, the approach should provide exposure for placement of rigid fixation that will allow early range of motion and return to baseline function. Moreover, Schneider et al. recently described a medial approach and reconstruction technique for chronic coronoid defects with posteromedial instability [6].

## Surgical principle and objective

Simple, reproducible technique to expose the medial elbow via a medial epicondyle osteotomy that provides excellent visualization of the ulnohumeral and proximal radioulnar joint, the anterior aspect of the coronoid and the proximal ulna and distal humerus. Most suitable for fixation of coronoid fractures, especially for complex fracture patterns like O’Driscoll anteromedial facet and basal fractures, delayed presentation of fractures, and revision surgeries.

## Advantages

**Fig. 1:**
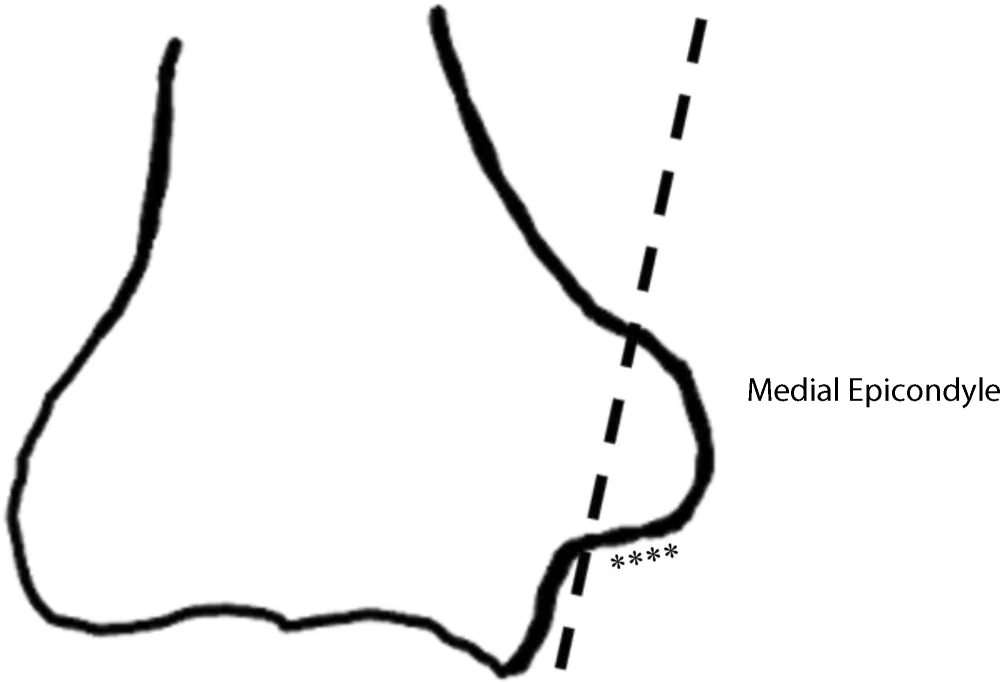
Author’s drawing of an anteroposterior view of the distal humerus with a dashed line indicating the proposed trajectory of a medial epicondyle osteotomy that preserves the medial ulnar collateral ligament (MUCL) origin, which is indicated by the asterisks

- The flexor pronator mass originates on the medial epicondyle, so an osteotomy effectively elevates the humeral head of the pronator teres, flexor carpi radialis, palmaris longus, humeral head of the flexor carpi ulnaris, and humeral head of the flexor digitorum superficialis together as a single sleeve of tissue [7]
- The medial ulnar collateral ligament (MUCL) originates entirely on the inferior surface of the medial epicondyle, with no contribution coming from the medial aspect of the trochlea [8]
- An osteotomy of the entire medial epicondyle that is parallel to the medial trochlea in the coronal plane also completely elevates the MUCL, while preserving its natural insertion on the bone (Fig. 1).
- The MUCL and flexor pronator mass are reflected together from proximal to distal. The Hotchkiss over the top interval anterior, and the FCU (Flexor carpi ulnaris) interval or the Taylor and Scham posterior can be used to extend depending on the structures to address.
- Extensive soft tissue releases are not necessary, thus, minimizing potential instability to the medial elbow.
- Allows excellent exposure to the ulnohumeral joint, anterolateral aspect of the proximal ulna, and articular surface of the coronoid.

## Disadvantages

- The exposure is limited anterior and posterior by the insertion of the intact MUCL on the proximal ulna at the sublime tubercle, which serves as a distal tether of this elevated sleeve of tissue.

## Indications

- Displaced fractures of the coronoid (particularly anteromedial and basal fractures)

## Contraindications

- Infected or compromised skin on the medial elbow
- Tear of the anterior bundle of the medial ulnar collateral ligament from the humeral attachment

## Patient information

- General surgical and anesthetic risks
- Potential nerve injury (ulnar nerve, cutaneous nerves)
- Elbow instability
- Implant-related complications (symptomatic hardware, hardware removal, hardware failure, repeat fracture)

## Preoperative work-up

- Clinical exam, with particular focus on the ulnar motor and sensory exam, forearm sensory exam, and flexor pronator mass function
- Computed tomography (CT) with three-dimensional (3D) reconstruction is recommended to assess the fracture pattern and for preoperative planning

## Instruments

- Standard orthopedic instruments for upper extremity/elbow surgery
- Osteotome set (straight and curved, 8–15 mm)
- Sagittal saw
- Reduction clamps
- Kirschner wires (1.2 and 1.6 mm), screws (2.7 and 3.5 mm), headless compression screws (2.4–3.5 mm), (anatomic) coronoid buttress plates, mini and small fragment plates and screws (2.0–3.5 mm)

## Anesthesia and positioning

- General endotracheal anesthesia with muscle relaxation
- Supine position with a hand table
- Sterile tourniquet
- Fluoroscopy can enter either from the head (preferred), from the side (perpendicular to the patient), or from the foot

## Surgical technique

(Figs. 2, 3, 4, 5, 6, 7, 8, 9, 10, and 11).

**Fig. 2:**
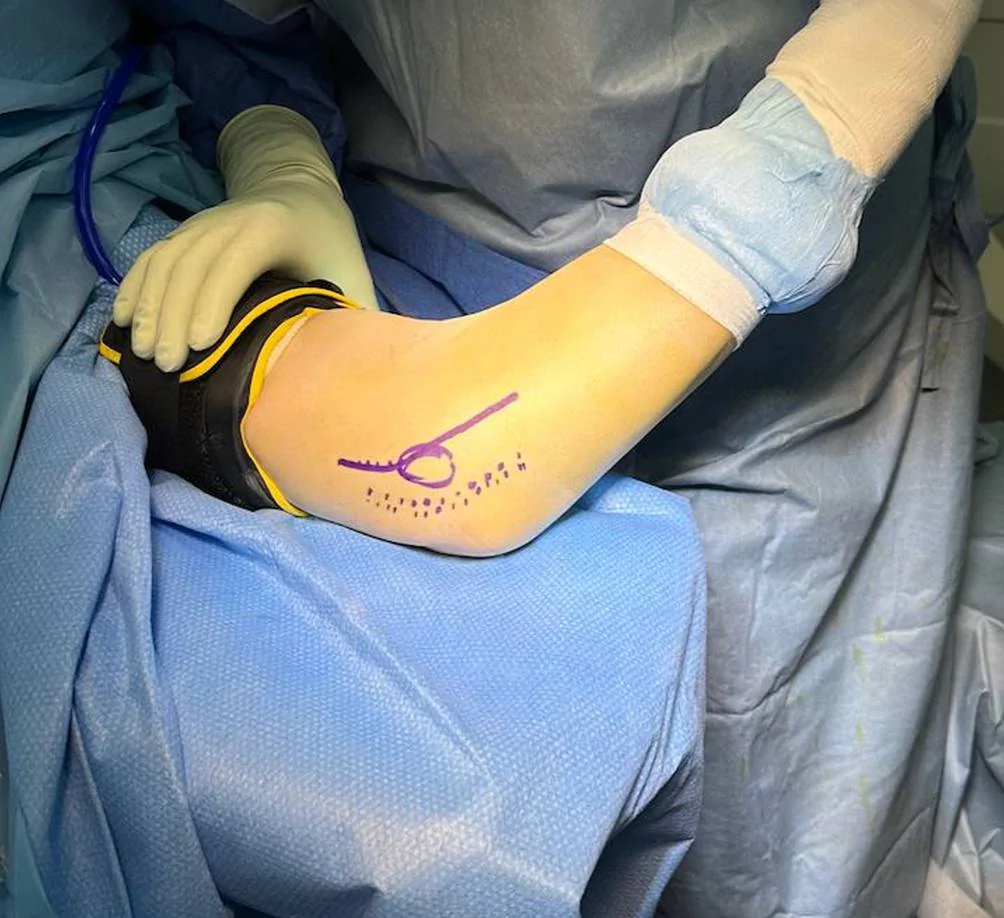
The incision is curvilinear, centered over the anterior aspect of the medial epicondyle. This is preferred so that the incision is not directly overlying the ulnar nerve, which is marked with dotted lines

**Fig. 3:**
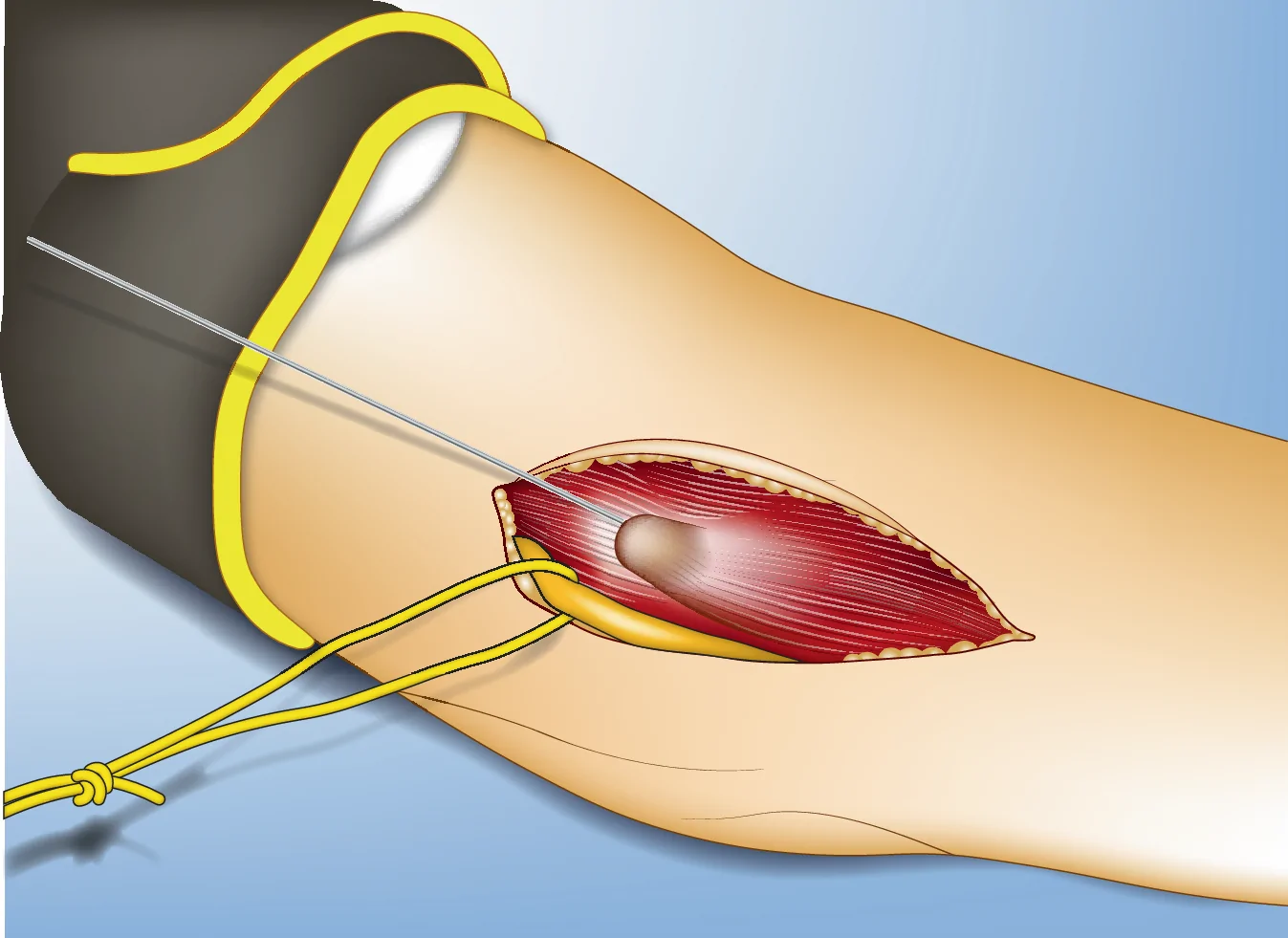
The ulnar nerve is identified and released in the cubital tunnel and between the two heads of the flexor carpi ulnaris. It is protected throughout the case. To plan the medial epicondyle osteotomy, a 1.2 mm Kirschner wire is first placed at the level of the proximal ridge of the lateral epicondyle and proximal to the flexor pronator mass into the ulnohumeral joint to serve as a landmark. A second 1.2 mm Kirschner wire is then placed along the medial supracondylar ridge of the distal humerus, from proximal to distal, and parallel to the medial trochlea and fibers of the flexor–pronator mass. This parallels the osteotomy

**Fig. 4:**
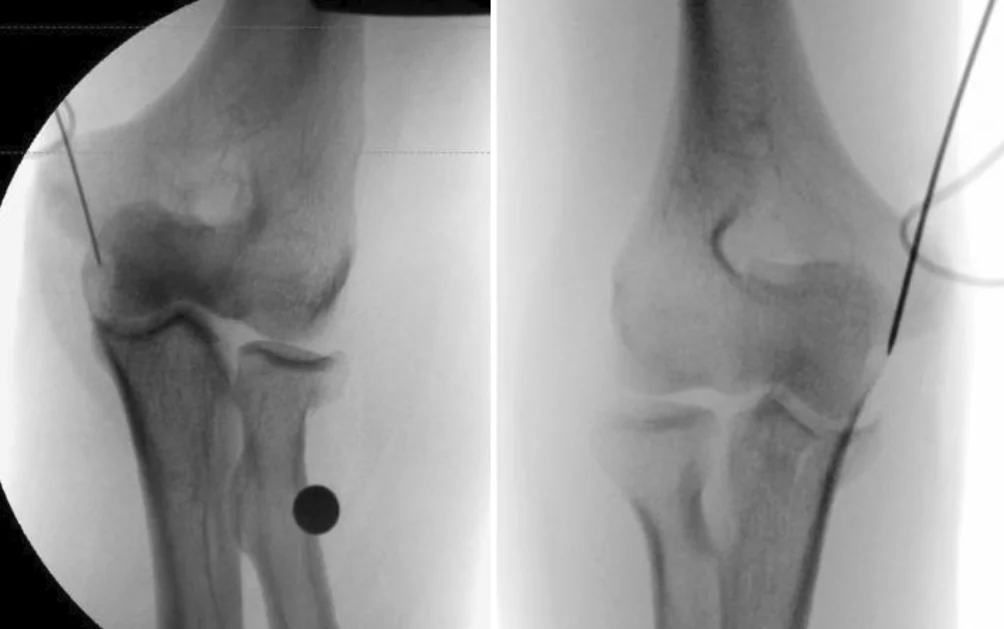
Fluoroscopy is used to confirm the trajectory of the Kirshner wire for a planned osteotomy that parallels the medial trochlea

**Fig. 5:**
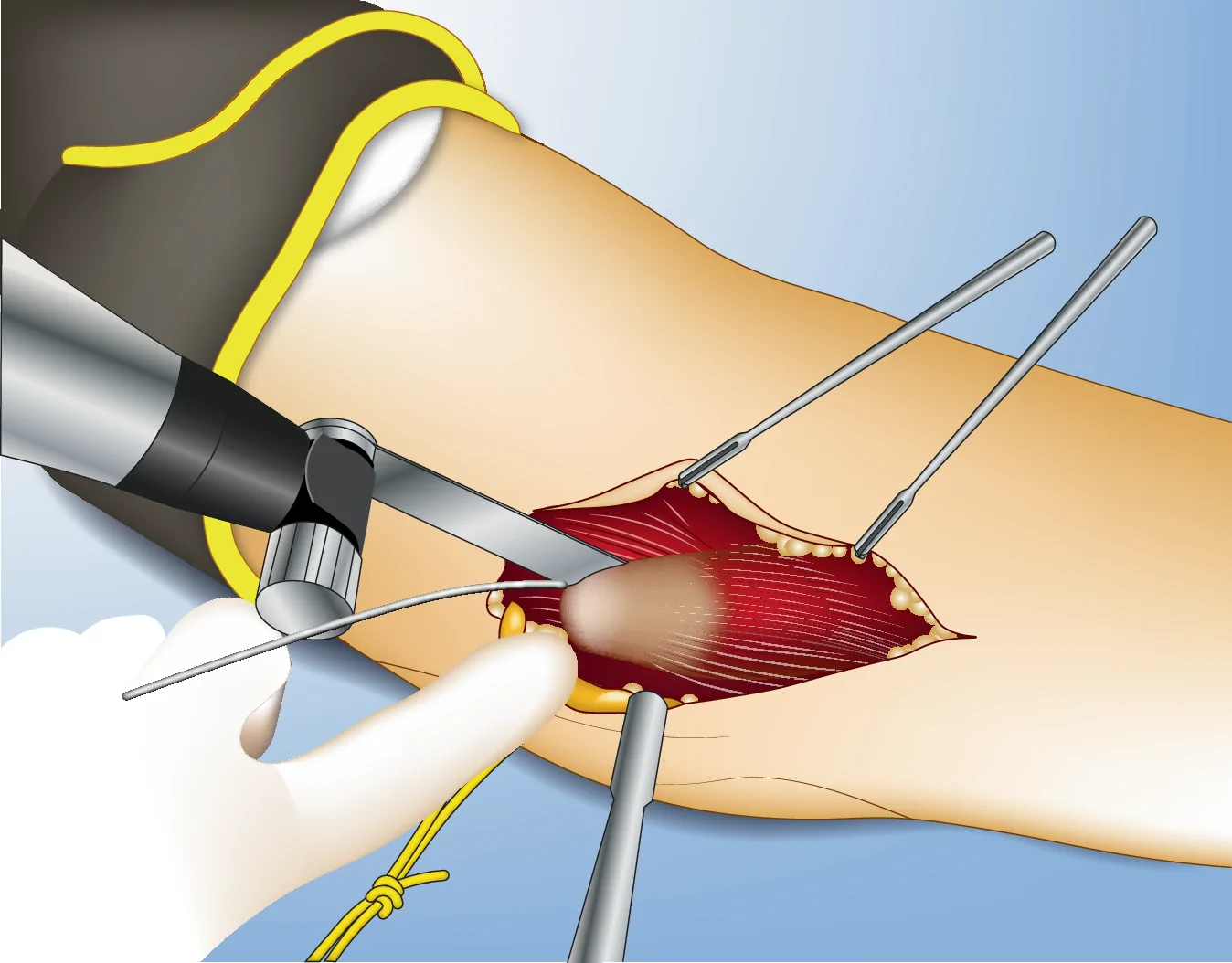
Hohmann retractors are placed posterior to the medial epicondyle and anterior to the ulnar nerve, carefully protecting the ulnar nerve from the osteotomy. While taking care to protect the ulnar nerve, a sagittal saw is used to perform the osteotomy, parallel to the medial trochlea and the fibers of the flexor–pronator mass, using the Kirschner wire as a guide

**Fig. 6:**
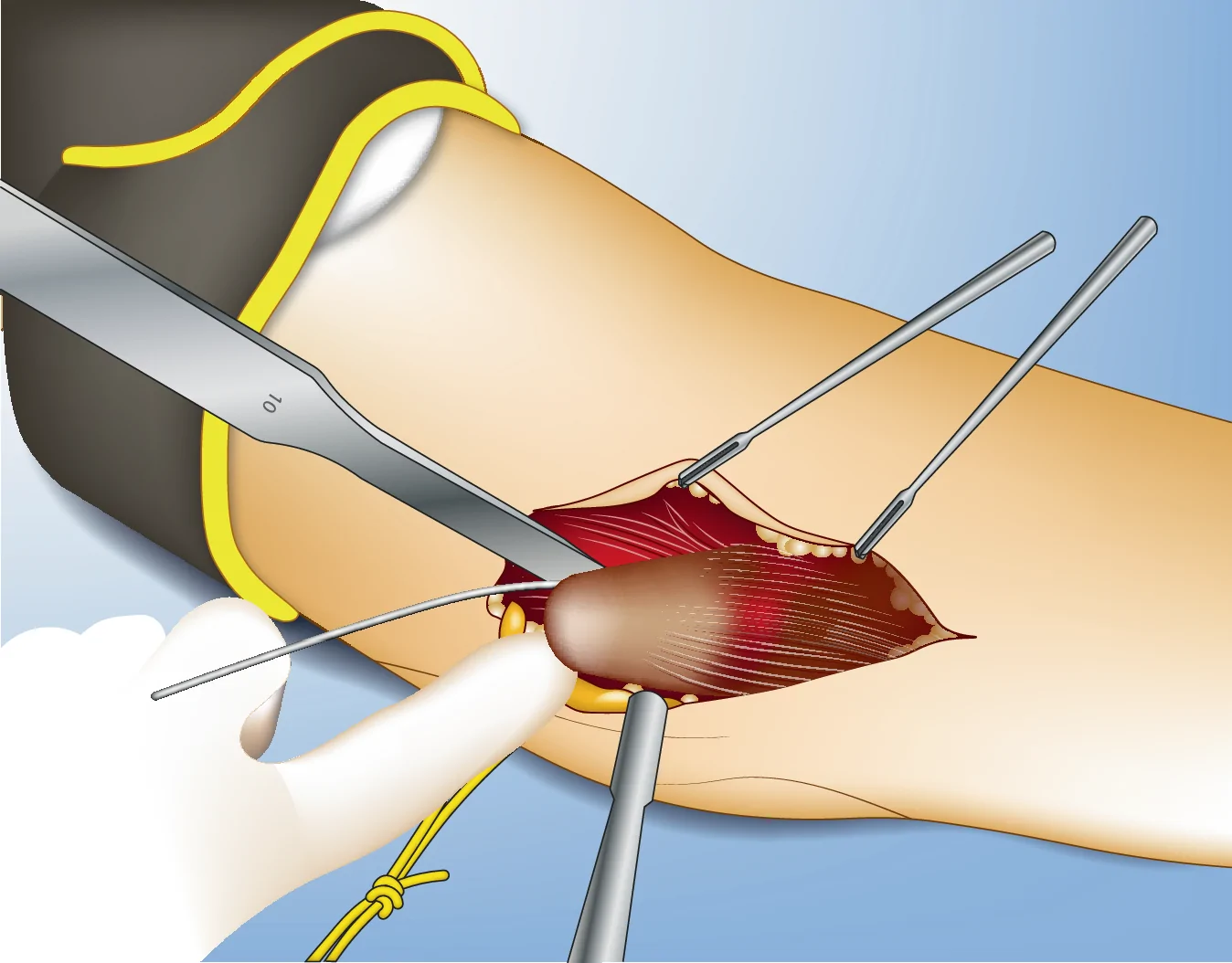
The osteotomy is completed with an osteotome. Completing the osteotomy with an osteotome instead of a saw helps to minimize soft tissue damage and creates a more favorable bone surface for healing

**Fig. 7:**
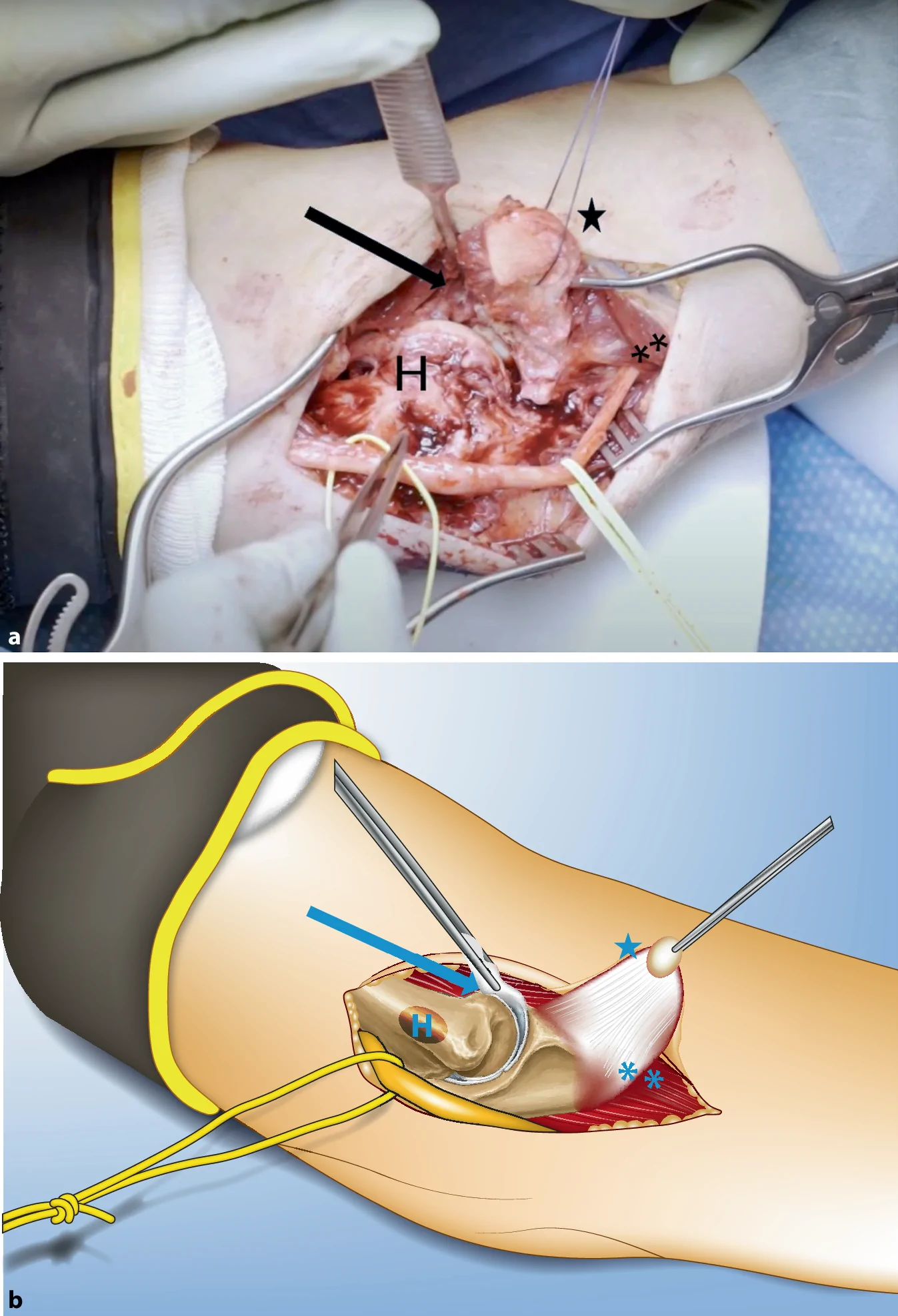
**a, b** The MUCL origin is reflected with the medial epicondyle, shown in this figure with the sutures (*star*). Minor soft tissue dissection of the anterior aspect of the flexor pronator mass may be necessary to complete the release at the level described by Hotchkiss in the Hotchkiss over the top approach. The anterior capsule is then released from the anterior humerus with an elevator (*arrow*), granting access to the ulnohumeral joint. If necessary, a posterior muscle split can be performed trough the FCU-interval (*asterisks*). (*H* humerus)

**Fig. 8:**
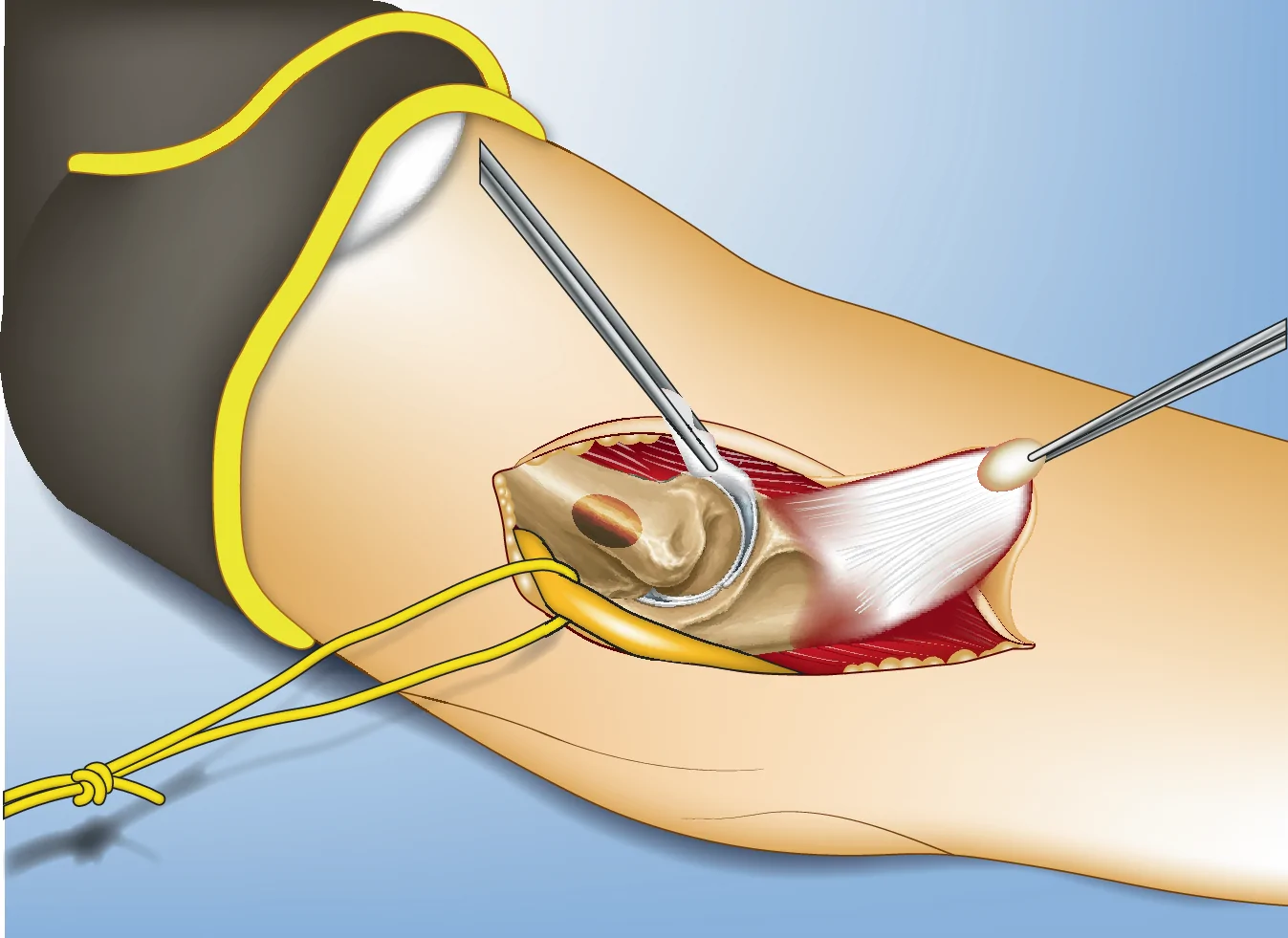
A gentle valgus stress together with the release of the medial ulnar collateral ligament and the flexor pronator mass opens the ulnohumeral joint from the medial side. The articular surface of the coronoid and fracture line are directly visualized

**Fig. 9:**
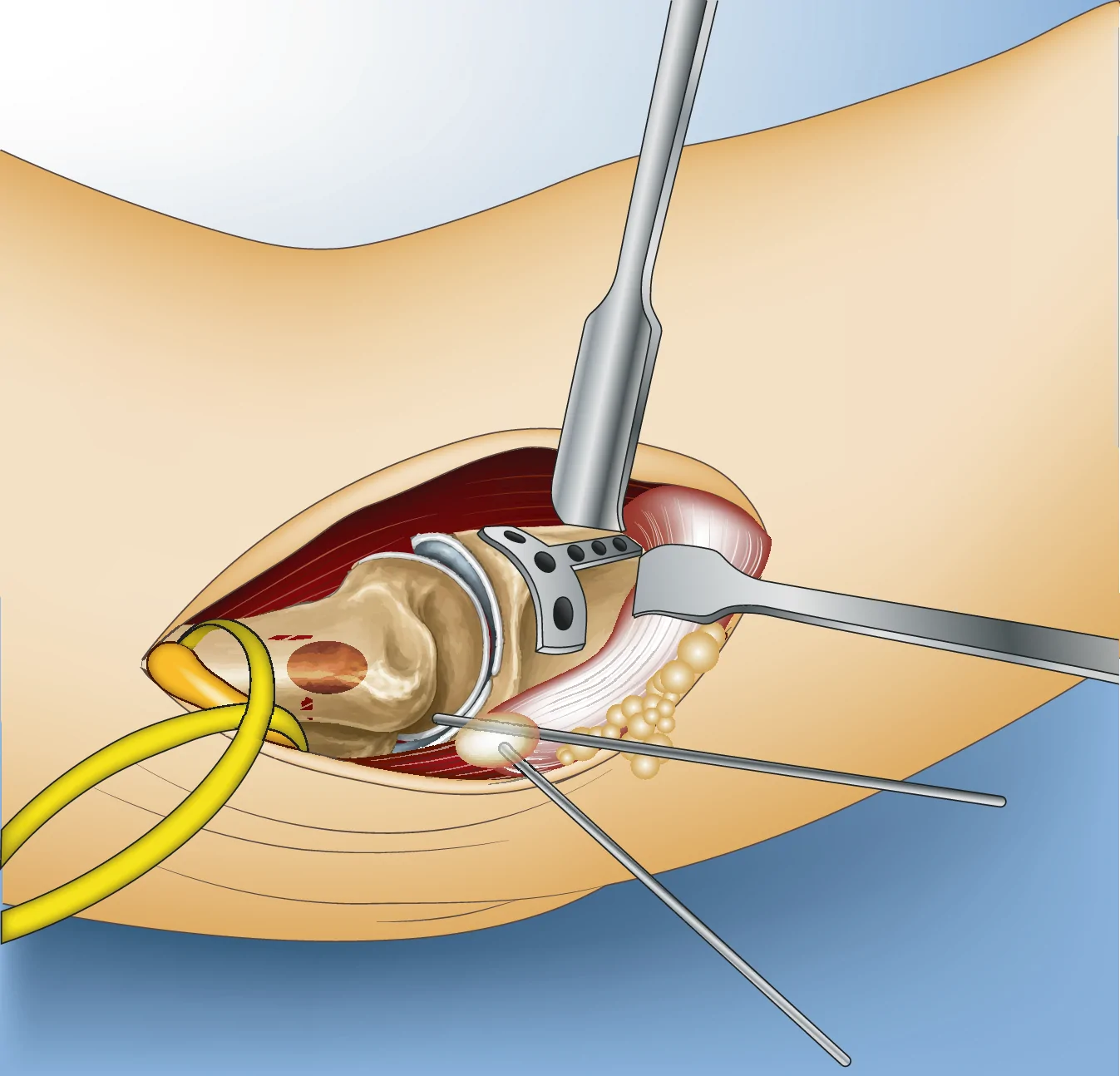
The approach also provides exposure of the proximal medial ulna for placement of a buttress plate. If more anterior or distal exposure of the ulna is required, the flexors can be split longitudinally, by splitting just anterior to the anterior border of FCU (Hotchkiss over the top), while being mindful of the median nerve

**Fig. 10:**
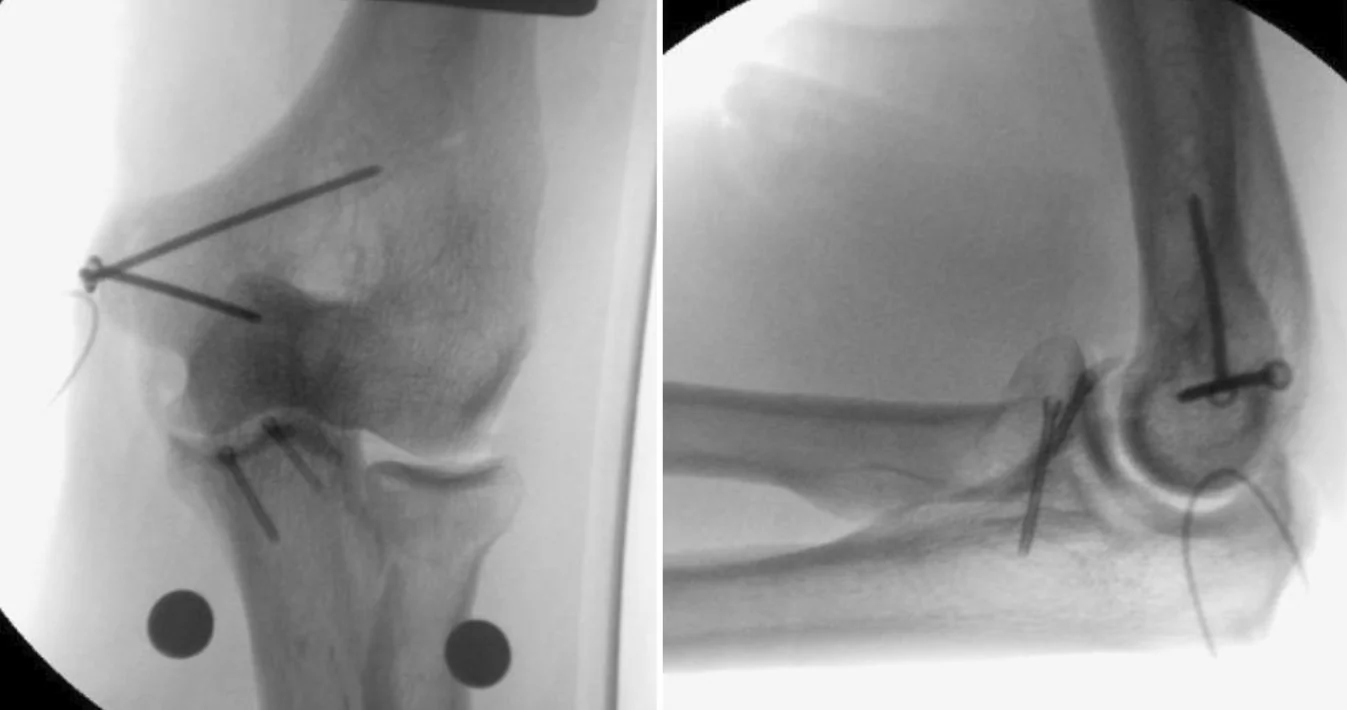
Provisional fixation followed by definitive fixation is then performed. The provisional reduction and fixation may be performed with Kirschner wires and clamps. Common definitive fixation constructs include headless compression screws (Fig. 10) or an anatomic buttress plate (Fig. 9), depending on the fracture pattern. For closure, the medial epicondyle is then reduced to its anatomical position and provisionally held in place with Kirschner wires or a clamp. For fixation of the medial epicondyle, two diverging 2.7 mm cortical screws are used, with one along the medial column of the distal humerus and another parallel to the joint. Alternatively, and at the surgeon’s discretion, the medial epicondyle screws can be predrilled prior to performing the osteotomy, but this is not mandatory. This is followed by layered closure of the soft tissue

**Fig. 11:**
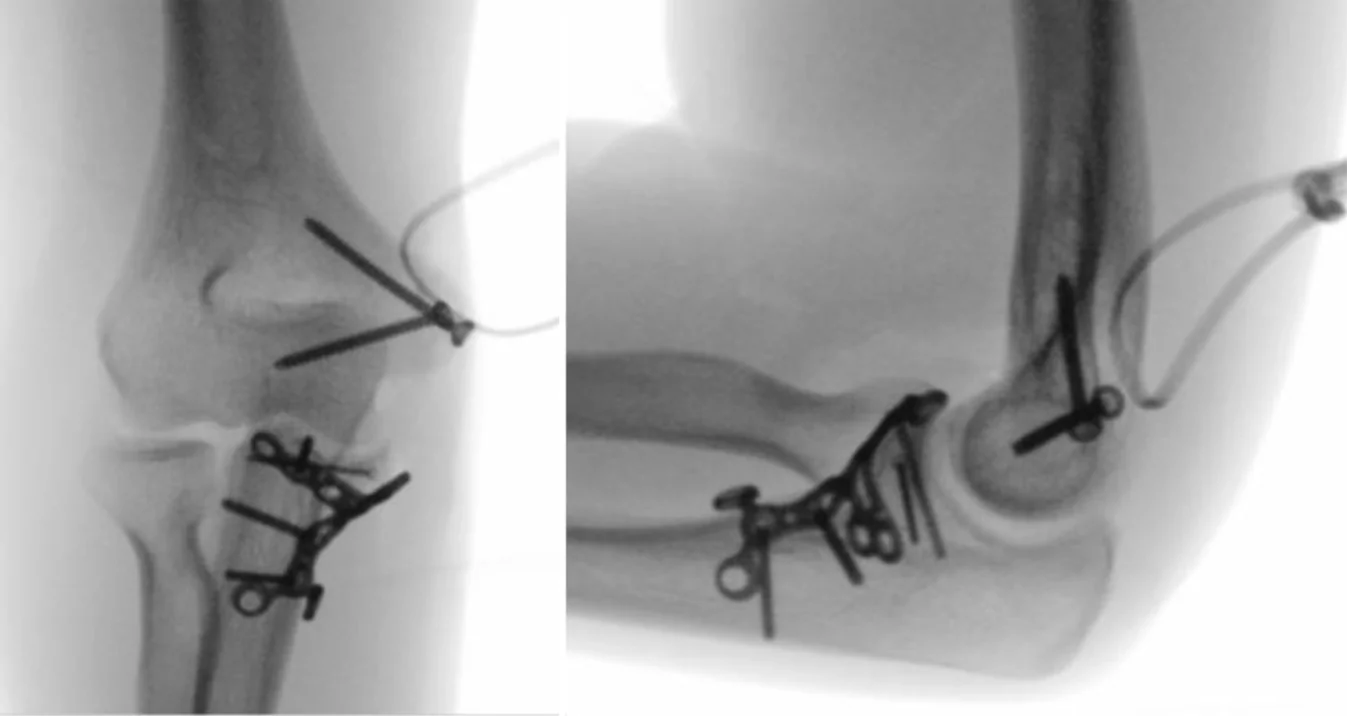
Fixation of the medial epicondyl osteotomy with 2.7mm cortical screws and coronoid fracture with headless compression screws

**Fig. 12:**
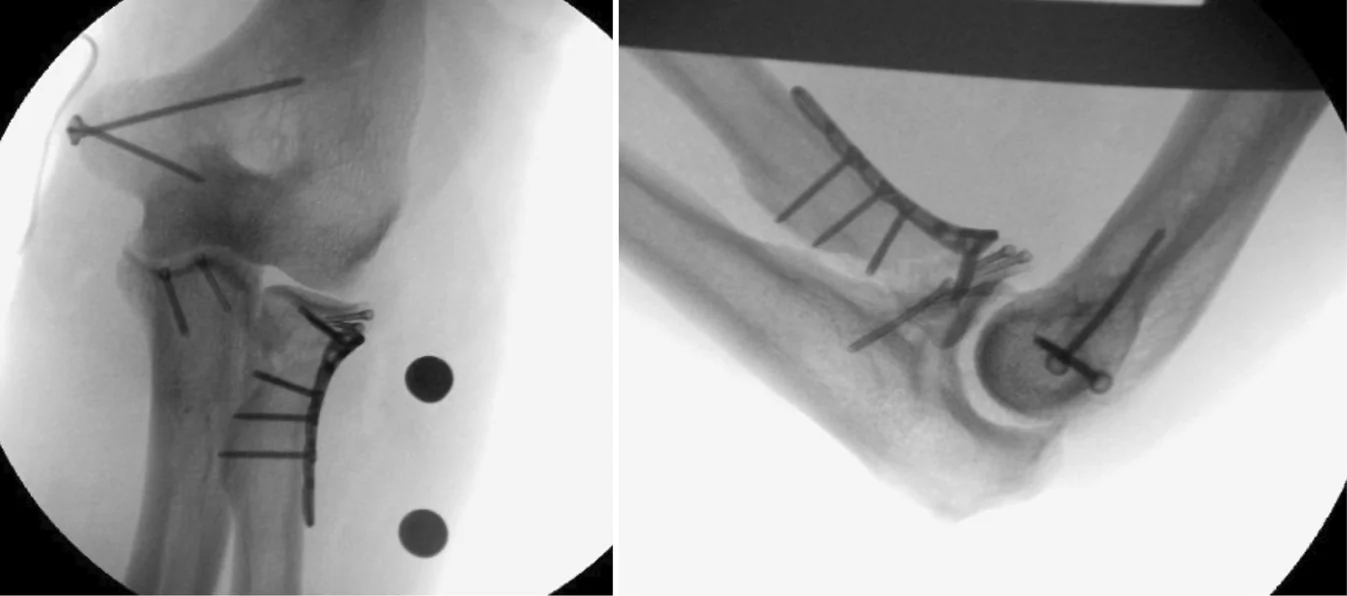
If there is pathology on the lateral side of the elbow, such as a radial head or neck fracture or injury to the lateral ulnar collateral ligament (LUCL) as in the case of a terrible triad injury, a separate approach should be performed to address the radius (lateral elbow approach is preferred)

## Special surgical considerations

With a subluxation event, the injuries may involve fracture of the anteromedial coronoid facet, injury to the lateral ulnar collateral ligament (LUCL), and injury to the posterior bundle of the medial collateral ligament (pMUCL). When this is the case, the fracture should be stabilized with a buttress plate or screws and the LUCL should be repaired if indicated. The pMUCL is not routinely repaired. If the patient has persistent posteromedial rotatory instability after the fracture and LUCL have healed, a delayed reconstruction of the pMUCL may be performed, but this is not usually done in the acute setting at the time of fracture fixation.

## Postoperative management

If adequate intraoperative radiographs are obtained (anteroposterior and lateral elbow), formal postoperative radiographs are not required. The patient is placed in a sling for comfort. Range of motion exercises are initiated under supervision of a physiotherapist. Strength exercises are initiated 6 weeks postoperatively. Expected return to work can vary depending on the nature of the work, from a few days for desk jobs to 12 weeks for physically demanding labor.

## Errors, hazards, complications

- Ulnar nerve neuropraxia from excessive traction
- Symptomatic hardware from prominent screw heads on the medial epicondyle
- Screw perforation into the elbow joint
- Refracture of the medial epicondyle
- Elbow stiffness from a combination of fracture and surgery

## Results

**Fig. 13:**
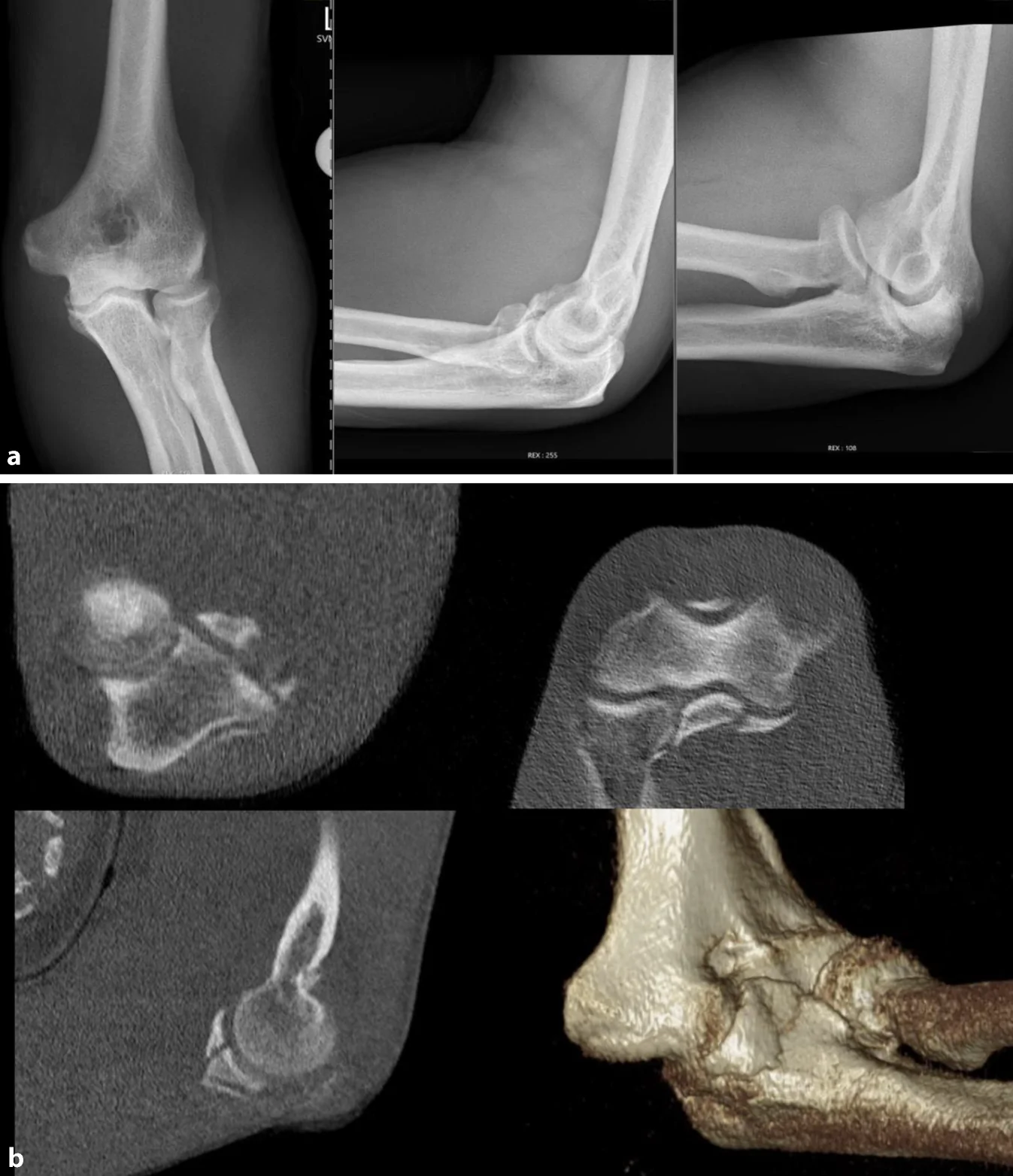
Radiographs (**a**) and computed tomography (**b**) demonstrate an O’Driscoll anteromedial type 2 fracture involving the anteromedial rim and tip. There is also a fracture of the radial neck. The final fixation construct is demonstrated in Fig. 12

**Fig. 14:**
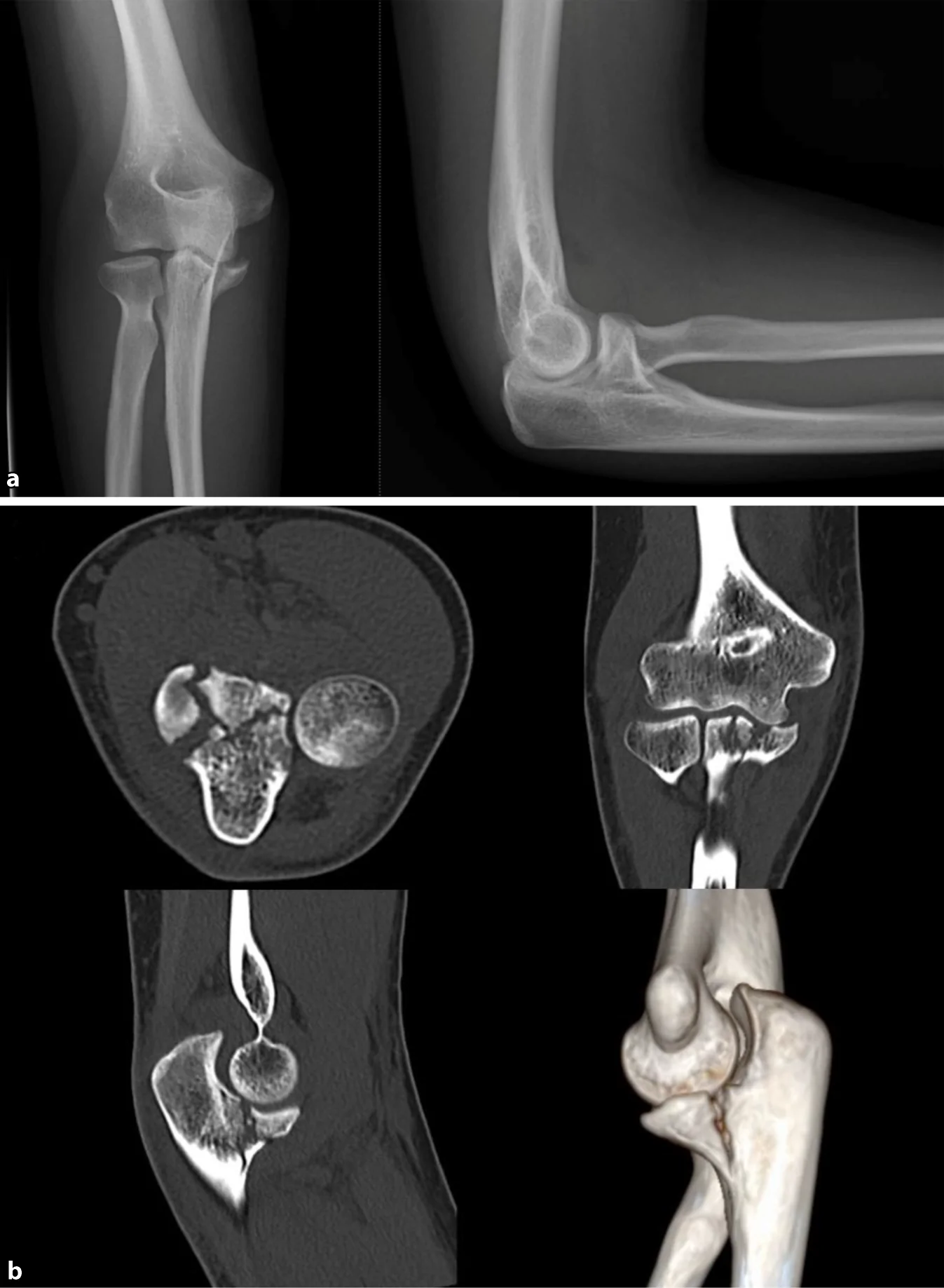
Radiographs (**a**) and computed tomography (**b**) demonstrate an O’Driscoll basal type 1 fracture of the coronoid body and base with extension into the lesser sigmoid notch. After fracture debridement and provisional fixation, a lag screw and buttress plate were selected to fix the multifragmentary coronoid base fracture (Fig. 11)

We describe 13 cases which were treated with this surgical approach done at two different hospital centers (Table 1, Figs. 13, 14, 15, 16, and 17). The patients were 7 men and 6 women. Ages ranged from 20–62 (mean 37.2) years. Fractures were either anteromedial or basal on the coronoid. All patients underwent open reduction and internal fixation of the coronoid through a medial epicondyle osteotomy. Fixation was performed with either headless compression screws or a buttress plate depending on the fracture pattern. Average follow-up was 14.1 months. One patient, who was a tourist and returned to their home country, was lost to follow-up.

**Table 1:** Patient demographics and postoperative elbow range of motion.

Patients	–	–	13
	Female	–	6
Age in years	–	–	37.2 (20–62)
Follow-up in months	–	–	14.1
Elbow range of motion			
–	Flexion	–	130° (90–155°)
	Extension	–	12° (0–45°)
	–	Arc of motion	118° (50–155°)
	Pronation	–	77° (40–90°)
	Supination	–	77° (35–90°)
	–	Arc of motion	154° (75–180°)

**Fig. 15:**
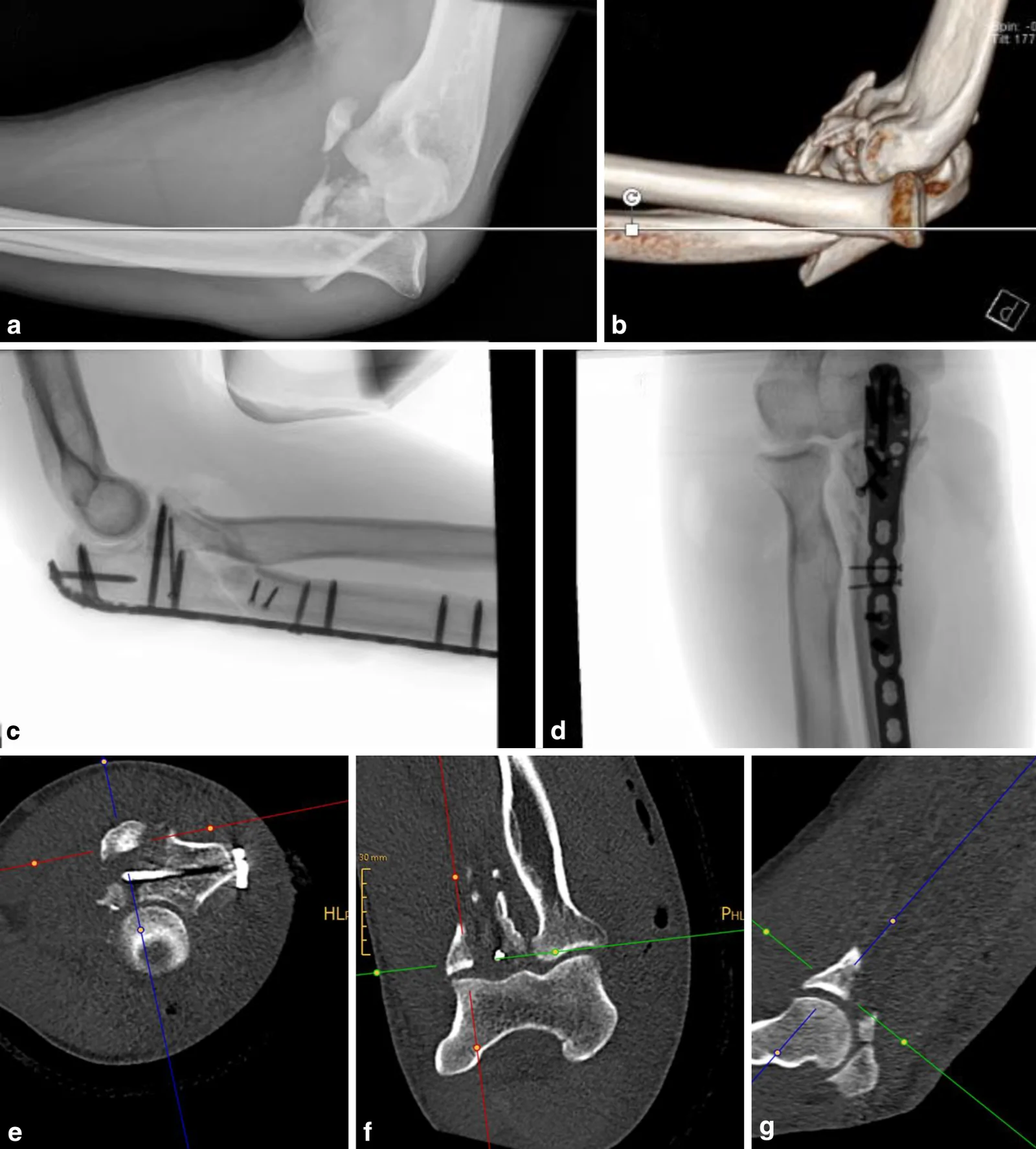
A comminuted Monteggia fracture (**a**, **b**) underwent proximal ulna fixation (**c**, **d**). Postoperative computed tomography demonstrated a malreduced coronoid (**e**–**g**)

**Fig. 15:**
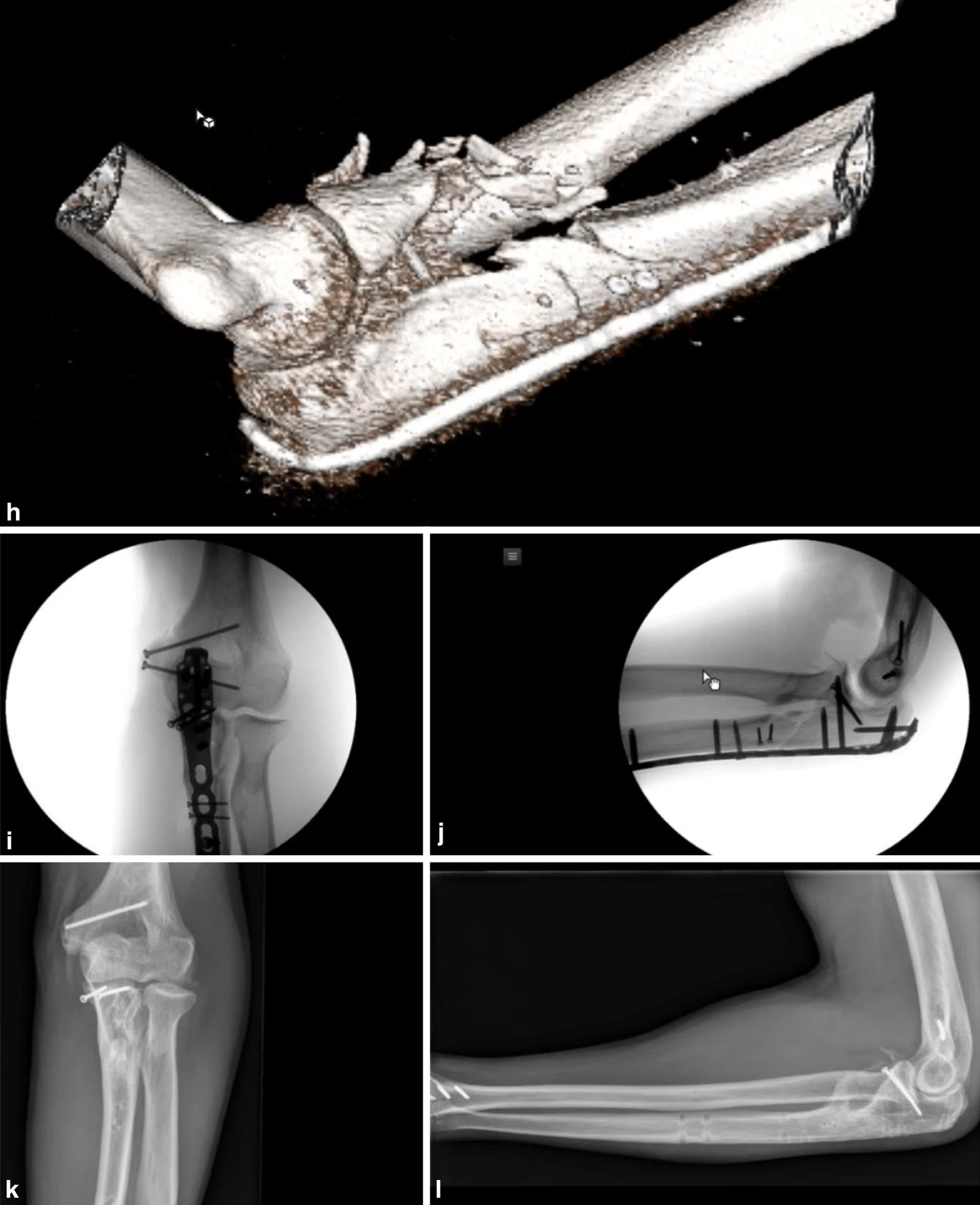
(Continued) Three-dimensional reconstruction (**h**) of the malreduced coronoid. Revision fixation of the coronoid with headless compression screws was performed through a medial epicondyle osteotomy (**i**, **j**). Hardware was later removed (**k**, **l**)

**Fig. 16:**
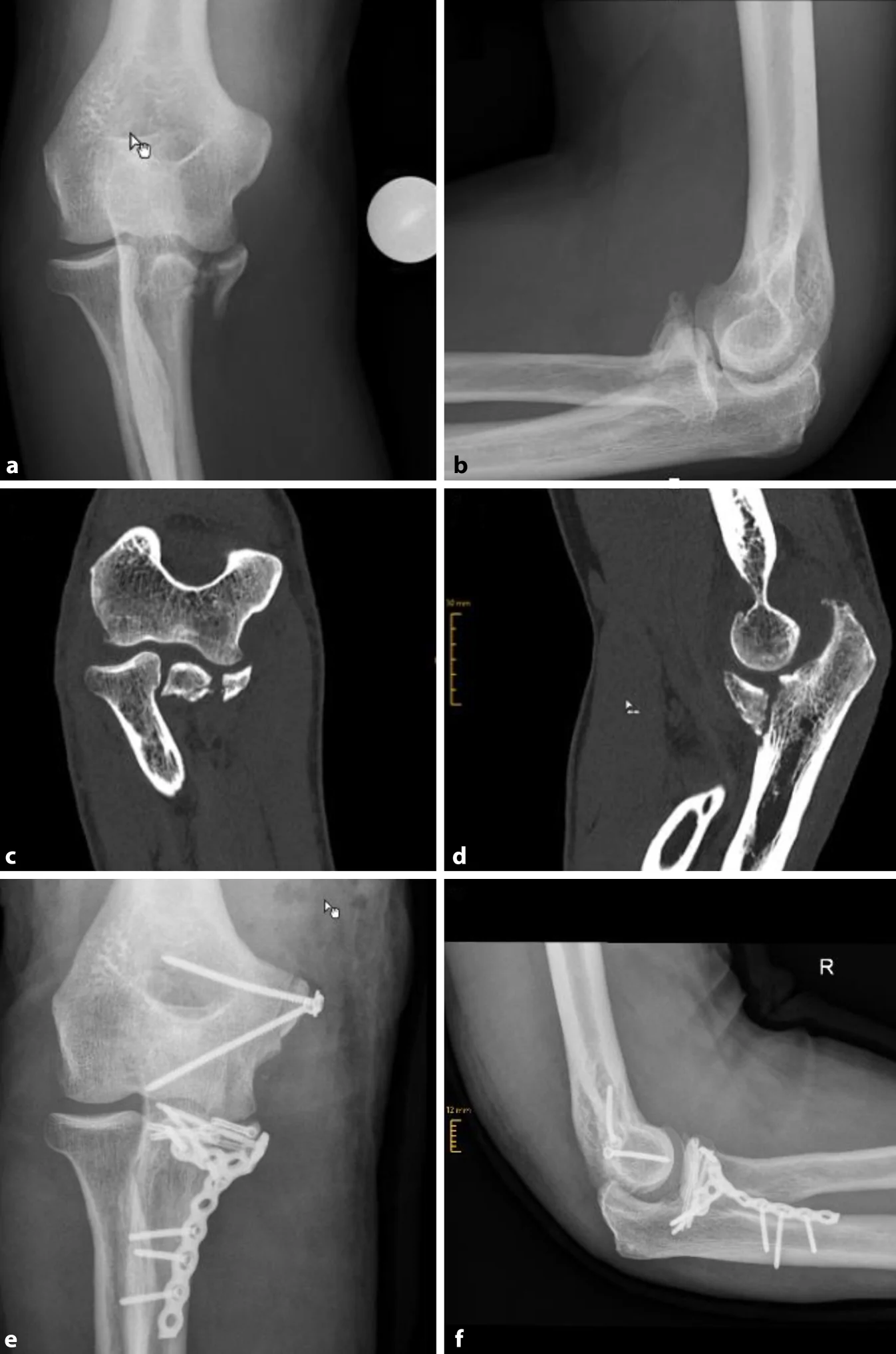
Radiographs (**a**, **b**) and computed tomography (**c**, **d**) demonstrate an O’Driscoll anteromedial type 3 fracture of the anteromedial facet and tubercle. Due to a delayed presentation, the patient underwent buttress plate fixation through a medial epicondyle osteotomy 7 weeks after trauma (**e**, **f**)

**Fig. 17:**
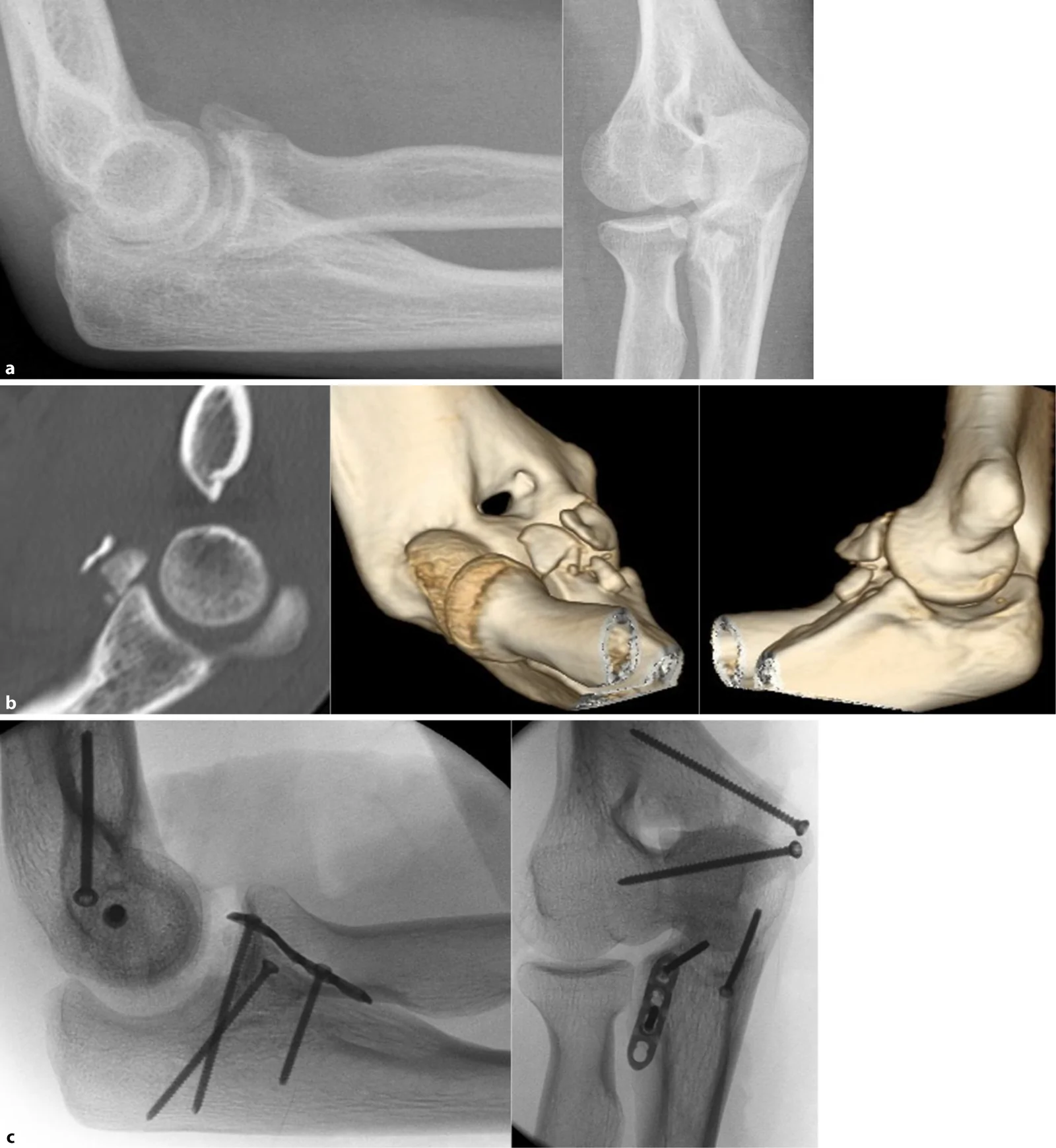
Radiographs (**a**) and computed tomography (**b**) demonstrate an O’Driscoll anteromedial type 2 fracture of the anteromedial rim and tip of the coronoid resulting in varus posteromedial rotatory instability. The fracture underwent fixation with a 2.0 mm buttress plate and an independent screw (**c**)

**Fig. 17:**
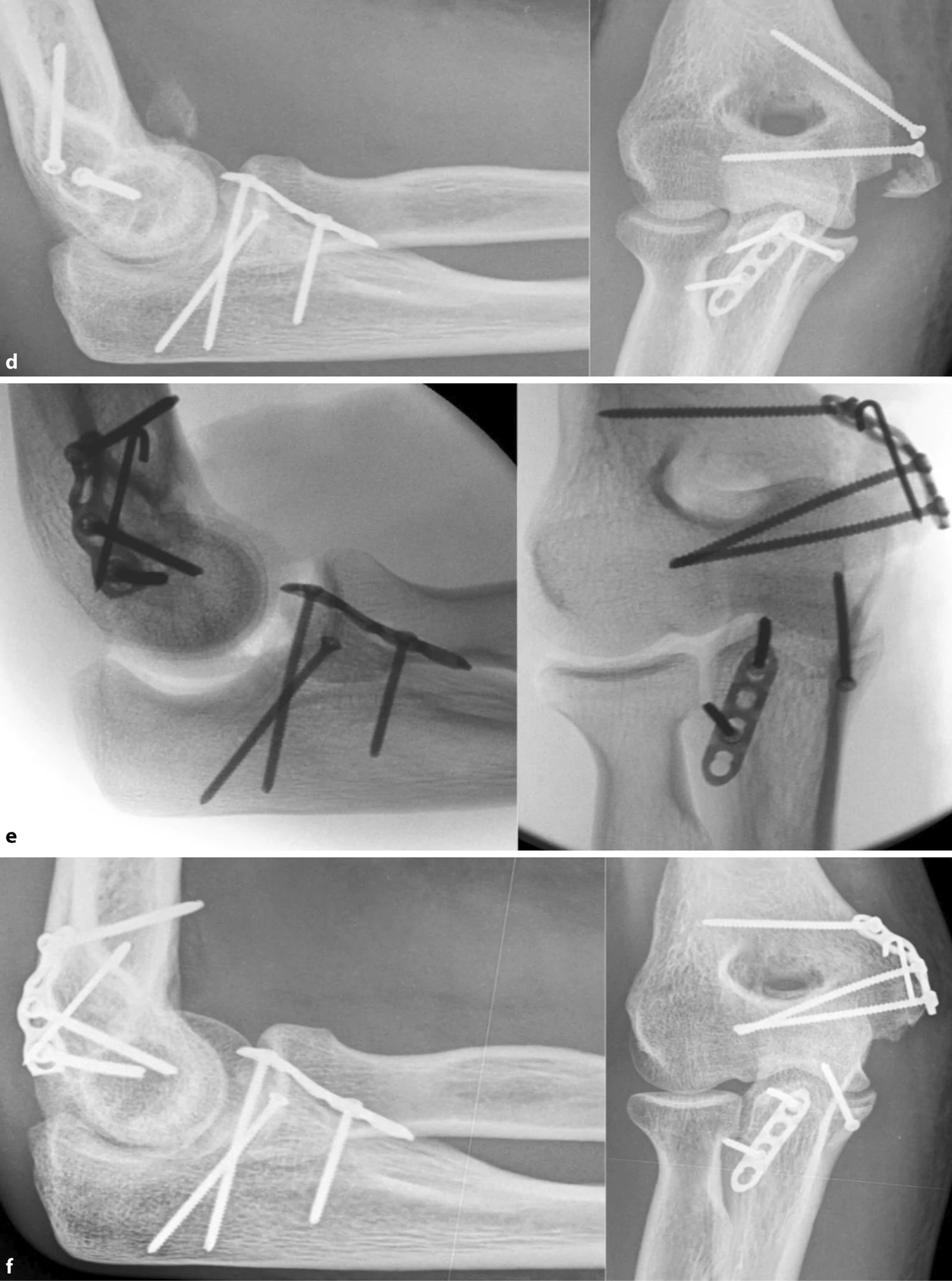
(Continued) Postoperative radiographs demonstrated loss of fixation of the medial epicondyle fragment (**d**). Revision fixation of the medial epicondyle was performed with a medial buttress plate. Intraoperative (**e**) and postoperative radiographs at 3 months (**f**) demonstrated stable fixation, with elbow range of motion from 155° of flexion to 0° extension and from 90° of pronation to 90° of supination

Average elbow range of motion was 130° of flexion (range 90°–155°) to 12° of extension (range 0°–40°). Average flexion–extension arc of motion was 118° (50°–155°). Average pronation was 77° (range 40°–90°) and average supination was 77° (range 35°–90°). Average pronation–supination arc of motion was 154° (range 75°–180°). The ulnar nerve was intact in all patients. Six patients underwent delayed implant removal from the medial epicondyle. One patient sustained a postoperative complication where the medial epicondyle refractured from implant failure. He underwent revision fixation with a medial epicondyle buttress plate (Fig. 17). Retrospectively, the epicondyle osteotomy was not at the level parallel to the medial trochlea, as described in Fig. 3, leaving a bone fragment that was too small to accommodate two screws.

The advantage of the osteotomy and subsequent repair is that important static and dynamic stabilizers of the medial elbow—namely, the MUCL and the flexor pronator mass—remains intact and the exposure is not limited by the level of the selected muscular interval. With an intact insertion of the MUCL at the sublime ligament, it is possible to work around the insertion point to position stabilizing implants. Stable fixation of the medial epicondyle allows for early range of motion to prevent elbow stiffness. In our patient population, we counsel patients that they may undergo a removal of hardware if the medial epicondyle screws provide symptomatic irritation.

## Data Availability

De-identified patient data can be made available upon request.

## Declarations

All procedures and study protocols mentioned were in accordance with the ethical standards indicated in each case. No human or animal studies were performed by any of the authors of this article. All patients provided informed general consent to use their blinded data for study purposes.
